# The Obstetric Memoirs and Contributions of James Y. Simpson, M. D., F. R. S. E., Professor of Midwifery in the University of Edinburgh, Etc.

**Published:** 1856-01

**Authors:** 


ART. V.— *The Obstetric Memoirs and Contributions of James Y. Simpson, M. D., F. R. S. E., Professor of Midwifery in the University of Edinburgh, etc.* Edited by W. O. PRIESTLY, M. D., Edinburgh, formerly Vice-president of the Parisian Medical Society, and HORATIO R. STORER, M. D., Boston, U. S., one of the Physicians in the Boston Lying-in-Hospital, Members of the Medico-Chirurgical and Obstetric Societies of Edinburgh, etc., etc. Philadelphia: J. B. Lippincott & Co. 1855.

This is the first of two volumes made up of the fugitive papers of Prof. Simpson, some of which have already been widely published in American Journals, while others appear here for the first time. Drs. Priestly and Storer have been pupils, and are now warm admirers of the author. They undertook the collection and publication of these papers as a labor of love, and a certain cordiality and warmth of feeling are recognizable in the dedications and preface. First Dr. Simpson himself dedicates them to Retzius, of Stockholm, while Dr. Storer, in this edition, includes in his dedication a number of names of distinguished Americans, among which we are most pleased to find that of our good friend and former colleague, Austin Flint. This compliment, alone, to one whom we so highly esteem, would incline us to a favorable opinion of its merits.

We do not share in the regrets so often expressed that Dr. Simpson has not embodied his experience in a work on obstetric science generally. The ceremonial observances of a formal book, the green withes of tradition which always influence the "standard author," would have crippled and impaired that straightforward energy and enthusiasm which manifests itself in the rapid monograph, the "hurrygraph," written because the author had something to say.

We cannot analyze these papers *seriatim*, though, could we feel sufficient confidence in our own judgment of mooted points, we could hardly fill our pages with more pregnant matter. But Dr. Simpson's name is linked with too many innovations and improvements to pass with a mere word of approval or censure. There is something in every word he says which irresistibly calls for attention, and demands consideration. To a few of these favorite ideas we shall allude.

In 1844 Dr. Simpson proposed, in certain cases of placenta prævia, the extraction of the placenta before the child.

This measure is one of two things: either a great improvement or a reckless innovation. It should be borne in mind that it calculates upon the death of the child as a necessary consequence. The interests of the mother are not only paramount, but no attempt is made to compromise them for the safety of the child. Of course Dr. Simpson does not propose any such rule of action in cases where it can be avoided, but he sums up the difficulties and objections to the usual practice of turning, in such a way as to create great dissatisfaction with that resource.

The operation of turning is in itself dangerous to the child; the mother may bleed to death before turning is rendered possible by a dilatation of the os sufficient for the introduction of the hand; any effort to hurry the dilatation by the forced introduction of the hand may lacerate the uterus, (an accident more dangerous here than in other cases, owing to the more vascular condition of the os, the parietes being here permeated by the enlarged vessel developed to supply the placenta), and finally the delay requisite to the operation may prove fatal.

The question now arises: are these difficulties inevitably connected with the accident? And if they are not, will the substitute offered by Dr. Simpson compensate in increased safety to the mother, for the admitted and certain loss of the offspring?

The first question Dr. Simpson answers by statistics, and the answer to the second is also involved in these.

He collates from various sources the result in 654 cases of placental presentation. Of these, in 180 cases, that is one in every $3\frac{6}{10}$, the mother perished. These cases were variously managed, some by extraction of the child, some by rupture of the membrane, &c.

The occurrence of such an alarming per centage of fatality to the mother in this accident, indicates the urgent necessity for devising some more effectual management, if it be possible to do so. To show that it is possible, Dr. S. collects from different records the histories of 141 cases in which the placenta was expelled or removed before the child, of which 10 died, being in the ratio of 1 in 14.

The evidence in favor of the prior extraction of the placenta, he remarks, becomes still more striking when we examine the mode of death in the ten fatal cases. Omitting the detailed histories, we will simply state that four of the ten died several days after delivery, from causes evidently not connected with hæmorrhage. In three other of these ten cases, *the hæmorrhage ceased after the detachment of the placenta*, the mothers dying from flooding previous to that event. In the three remaining cases the death was chargeable to flooding subsequent to the detachment of the placenta.

Assuming, then, that these three latter cases are the only ones directly connected with the entire detachment of the placenta, we should have only *one* death in forty-seven cases where the detachment of the placenta preceded the delivery of the child, against one in three and six-tenths in the other cases.

If these statistics are accurate, if we can reasonably expect to save the mother in all but one in forty-seven cases of placenta prævia, it follows that we should, at once, in all cases where the hæmorrhage is sufficient to endanger the life of the mother, proceed to detach the placenta and thus sacrifice the child. We may remark in this connection that it would be interesting to know the per centage of children saved after turning.

Of course so important a change in our management of this accident should rest on some theory of the sources of the hæmorrhage. Dr. Simpson rejects, entirely, the universally acknowledged notion that the blood proceeds from the exposed maternal orifices in this accident. He supposes that the hæmorrhage is *from the placenta*: that is, that the separated or lacerated decidual veins are constantly receiving a supply of blood from the utero-placental vessels of the part still attached; that, accordingly, the hæmorrhage will increase with the amount of separation only to a certain limit, after which it will decrease, and that the whole placenta being separated the hæmorrhage will cease— *Why?* Because the "attractive power" of the placenta upon the uterine vessels is lost, a theory which has many facts in analogy to it.

Another reason might be adduced, though we have failed to find it in our author. The process of dilation of the os involves a *retraction* of its lips, which is equivalent to a *contraction*, and would tend to check hæmorrhage. This retraction would of course go on more rapidly after the detachment of the placenta.

We have given but a few of the leading arguments involved in Dr. Simpson's paper, leaving many minor but important details and analogies untouched.

Before passing to another of the subjects treated in this volume, we must say that Prof. Simpson has manifested the greatest fairness in his argument. The naked skeleton we have given, in his hands is clothed with a most praiseworthy minuteness of detail, both as to the authority whence it was derived, and in giving, in so many instances (in most of the more important) a careful history which enables us to estimate the features and peculiarities of the individual case.

The next and only subject mentioned in the volume, which we propose to notice here, is that of the *substitution of turning for craniotomy*.

The murderous operation of embryulcio, is one of those alternatives of obstetric practice in which the practitioner must choose between two evils, and bargain away one life for another. Such a compromise is ever painful. If any means can be devised to decrease the frequency of its necessity, and afford to the accoucheur a chance for saving both lives, it should be hailed with gratitude.

Certain objections to the proposed substitute at once arise. Having turned and delivered the feet, the head is still above the pelvis, as large as ever, and the brim is still as small. More than this, nature has indicated the cephalic position as the normal one, while this operation proposes to reverse that position, and choose one unnatural, though not classed among the dystociæ. The latter objection loses its force, however, when we reflect that the pelvis itself is here unnatural, and that a natural and well shaped head is as little adapted to it, as is a monstrosity to the well shaped pelvis. The relation of the two parts being destroyed, art is at liberty to substitute any other relation it may deem more advantageous. This is what Dr. Simpson claims to have done.

The general shape of the fœtus, he argues, is that of a cone; the base of the cone being formed by the bi-parietal diameter of the head. It follows that in natural labors the principle of the wedge is reversed, an apparent defect compensated by the fact that no pressure is necessary upon the child's head, in natural labor, until the occipital apex of the "cephalic cone" is sufficiently advanced to receive it, while, if the brim of the pelvis be much contracted, this apex of the wedge cannot be entered. The head—the only point of difficulty—is in itself a double cone. Of its two apices one is found in the occiput, the other—truncated—in the bi-mastoid diameter of the head. If the bi-mastoid diameter be first introduced into the brim of the pelvis, a steady lateral compression may be applied to the parietal diameter by which the parietal bones may be compressed, or even indented, so much as to permit the passage of the entire head, traction being made from the feet. This point Dr. S. illustrates by using the letter A. Let the letter A represent a round cone simulating the figure of an infant, the apex of the letter corresponding to the feet, the cross-bar to the bi-mastoid diameter, and the two diverging feet the elastic and compressible arch of the cranium. To carry out the illustration in his own language:

"Now if we desired to pass this round cone A through an oblong aperture O, the diameter of which was somewhat *less* than the diameter of the basis of the cone, should we succeed best by pushing it through the oval opening with its basis foremost, or by dragging it through its apex foremost? If we extract the cone through the aperture by bringing its narrow end foremost—or, in other words, is we bring the child by the feet instead of the head — then two objects are gained: for, first, we have the power of using any justifiable degree of force that may be required, by the command we thus obtain of the protruded and narrow end of the cone; and, secondly, the elastic sides of the base of the cone situated above the cross-bar — or, in other words, the sides of the cranium itself above its basis — will yield and become compressed together to such an extent as to enable the collapsed body to pass through the supposed opening."

This is the argument, or, at any rate, a part of it. To us the proposal seems feasible, but it is still a question *sub judice*. It is, however, supported by a long train of able reasoning, and a large draft upon the author's wide experience. Few minds can read it without yielding credence, but even after that it will require some courage to resort to the operation.

In passing from the consideration of this book we cannot too earnestly commend it to the attention of our renders. The mind may be convinced, or may dissent, from the author's views; but it cannot come from their perusal unrefreshed or uninstructed. The earnest, laborious, practical thinker shines out on every page; and the industry which we know to be the character of the man is evident in every subject treated.

Dr. Simpson is now the leading obstetrician of the world. Through many startling innovations on established practice; through some personal strifes in which he has been as earnest and successful as elsewhere; and through some — not few nor unimportant — positive additions to obstetric and medical science, his name has become a household word wherever his language is spoken. We admire the indomitable energy and the spirit with which he demolishes his enemies, almost as much as his really splendid abilities. But he is an honestly successful man; such an one as we never envy, and would always emulate.

